# Breastmilk cadmium levels and estimated infant exposure: a multicenter study of associated factors in a resource-limited country

**DOI:** 10.1186/s13006-023-00574-0

**Published:** 2023-07-28

**Authors:** Ramzi Shawahna, Rana Saleh, Lina Owiwi, Aya Abdi, Diana Bani-Odeh, Iyad Maqboul, Hatim Hijaz, Mohammad Jaber

**Affiliations:** 1 Department of Physiology, Pharmacology and Toxicology, Faculty of Medicine and Health Sciences, An-Najah National University, New Campus, Building: 19, Office: 1340, P.O. Box 7, Nablus, Palestine; 2 Clinical Research Center, An-Najah National University Hospital, Nablus, 44839 Palestine; 3 Department of Medicine, Faculty of Medicine and Health Sciences, An-Najah National University, Nablus, Palestine; 4 An-Najah National University Hospital, Nablus, 44839 Palestine

**Keywords:** Cadmium, Breastmilk, Inductively coupled plasma mass spectrometry, Lactation, Women

## Abstract

**Background:**

Despite the undisputed benefits of breastfeeding, infants might become exposed to xenobiotics that could be excreted into breast milk following maternal exposure. This study was conducted to assess breastmilk cadmium levels among lactating women in Palestine, a resource-limited country. Estimated daily intake (EDI) of cadmium via breastmilk was also calculated and predictors of high breastmilk cadmium levels and high infant exposure via breastmilk were identified.

**Methods:**

This multicenter study was conducted using a descriptive-analytical design. The lactating women were recruited from different maternity and public health clinics in all regions of Palestine. Demographic variables and exposure to sources of cadmium were collected in an interviewer-administered questionnaire. Foremilk samples (about 5 mL) were collected in polyethylene tubes using the hand-expression technique. The breast milk samples were collected in the period between December 2020 and March 2021. A pre-validated method using inductively coupled plasma mass spectrometry (ICP-MS) was used to quantify breastmilk cadmium levels. EDI values were calculated from the quantified breastmilk cadmium levels.

**Results:**

Breastmilk samples were obtained from 256 lactating women. The mean breastmilk cadmium level was 0.34 (SD: 0.33) μg / L and the mean EDI of cadmium via breastmilk was 0.059 (SD: 0.058) µg / kg per body weight / day. Breastmilk cadmium levels were quantified in 92.6% of the breastmilk samples. Of the breastmilk samples, 13 (5.1%) had cadmium levels above those reported as “normal” by the World Health Organization (WHO). Multiple linear regression showed that higher breastmilk cadmium levels and higher EDI were predicted by being a smoker, living in a refugee camp, living close to an industrial area, living close to disposal of wastes, living close to paint shops, living in a house with peeling / chipping paint, frequent use of cosmetics, frequent use of hair dyes, and not using vitamins.

**Conclusion:**

The breastmilk cadmium levels and infant exposure were predicted by maternal exposure to sources of cadmium. The findings reported in this study are valuable to antenatal and postnatal healthcare service providers. More studies are needed to plan and implement measures to reduce breastmilk cadmium levels and infants’ exposure to cadmium via breastmilk.

**Supplementary Information:**

The online version contains supplementary material available at 10.1186/s13006-023-00574-0.

## Background

Breastmilk is the optimal nutritional source for neonates [1]. Breastmilk is known to contain the necessary elements for normal growth and development including proteins, lipids, carbohydrates, and many other biologically active elements. Therefore, different health organizations and professional associations have recommended women to exclusively breastfeed their infants for the first six months of life followed by the gradual introduction of other foods along with continued breastfeeding until the age of two or beyond [2, 3]. Despite the undisputed benefits of breastfeeding, infants might become exposed to xenobiotics that could be excreted into breast milk following maternal exposure. Several heavy metals including lead, cadmium, mercury, and arsenic were previously reported among the harmful xenobiotics that infants might become exposed to via breastmilk [4, 5].

Human exposure to environmental contaminants has been escalating lately as a result of the fast-paced urbanization and industrialization across different regions of the world. Cadmium is one of the top toxic heavy metals listed in the priority list of hazardous substances of the Agency for Toxic Substances and Disease Registry (ATSDR) [6]. Cadmium was shown to be emitted into the soil, water, and air as a result of metal mining, refining, use of fertilizers, fossil fuel burning, disposal of wastes, and incineration [7]. Therefore, cadmium was reported to have contaminated crops, meat and meat products, and aquatic organisms [8]. Tobacco smoking is another commonly reported source of exposure to cadmium [9]. Cadmium was also classified as a Class I carcinogenic by the International Agency for Research on Cancer (IARC) [10]. Previous studies have reported that exposure to cadmium could be associated with kidney, lung, pancreas, and prostate cancers [11]. Acute exposure to high cadmium sources can cause immediate irritation in the stomach, vomiting, diarrhea, and even death. On the other hand, chronic exposure to low cadmium sources over a long period would lead to cadmium buildup in the kidneys and bones [11]. This would lead to kidney failure, demineralization of bones, and increased risk of fractures [12]. In infants, the adverse effects of exposure to cadmium were mainly in the renal, hepatic, cardiovascular, and nervous systems [13]. These adverse effects were shown to harm the growth, development, and overall health of the exposed infants. Prenatal and early infant exposure to cadmium was shown to impair cognitive functions and induce neurodevelopmental abnormalities [13, 14].

Small amounts of cadmium were reportedly found in breast milk obtained from lactating women in different regions of the world [4, 15]. The World Health Organization (WHO) recommended that breastmilk cadmium levels should be less than 1 μg / L [15, 16]. Previous studies were conducted to assess cadmium levels in breast milk and other biological fluids [17]. However, few studies aimed to determine the associations between demographic and other variables of lactating women with breastmilk cadmium levels. Additionally, little was reported on the variables that could be used as predictors of high breastmilk cadmium levels among lactating women in developing and resource-limited countries. Therefore, this study was conducted to assess breastmilk cadmium levels among lactating women in Palestine as a resource-limited country. These levels were used to calculate theoretical daily doses of cadmium that infants would receive via breastmilk. The other objectives were to determine the associations and identify the predictors of high breastmilk cadmium levels and high exposure.

## Methods

### Study design

This multicenter study was conducted using a descriptive-analytical design. The study was conducted and reported in adherence to the Strengthening the reporting of observational studies in epidemiology (STROBE) guidelines [18].

### Study population and sampling

The study population in this study was lactating women who visited eight maternity and public health clinics in different regions of Palestine for routine postnatal healthcare services. The sample size needed for this study was calculated at a 95% confidence interval (95% CI) based on Eq. (1):

1 $$Sample size (n)= \frac{{z}^{2}{\sigma }^{2}}{{D}^{2}}$$

The value of Z at 95% CI was 1.96; σ was the standard deviation (SD) of cadmium in breastmilk as reported in previous studies. In this study, σ = 1.18 was obtained from the study of Bassil et al. [19]. The precision (D) was set at 0.1.

The lactating women were invited to take part in this study by field researchers who visited the maternity and public health clinics, explained the study objectives to potential participants, and obtained their written informed consent.

In this study, the lactating women were included if they met the following inclusion criteria: 1) a healthy breastfeeding woman; 2) absence of chronic diseases; 3) had a term delivery; 4) expressed willingness to respond to items in a questionnaire and provide a breastmilk sample of about 5 mL; and 5) provided written informed consent.

### Data collection

The lactating women who agreed to take part in the study were asked to sign a written informed consent. The women also responded to items in a questionnaire that was developed based on previous studies that investigated heavy metals in breast milk [19–22]. The questionnaire collected the demographic variables of the lactating women like age, number of children, age of child, employment status, place of residence, household monthly income, and educational status. The lactating women were also asked about potential exposure to cadmium such as smoking status, living in the vicinity of an industrial area (living within 500 m of an industrial area was defined as near) [20, 22]; living in the vicinity of disposal of wastes (living within 500 m of disposal of wastes was defined as near); distance to the closest paints shop (living within 500 m of a paints shop was defined as near); living in a house with peeling or chipping house paint; frequency of using cosmetics (frequent use was defined by reporting daily or almost daily use); and use of hair dyes (frequent use was defined by reporting usual use). The lactating women were also asked about the frequency of using vitamins (frequent use was defined by reporting usual use, i.e., daily or almost daily). The questionnaire is provided as Supplementary Table S1.

To collect breastmilk samples, the lactating women were provided with heavy metal-free polyethylene tubes and were asked to hand-express about 5 mL of foremilk. Before expressing, the lactating women were asked to clean their hands and chest area using wet wipes that were soaked in isopropyl alcohol. The breast milk samples were collected in the period between December 2020 and March 2021. The foremilk samples were shipped to the laboratory at 4 °C and were stored at –20 °C until the time of analysis.

### Analytical procedure

All chemicals and reagents were purchased from Sigma-Aldrich (Darmstadt, Germany). All tubes and glassware were incubated overnight in 10% HNO_3_ to prevent the adsorption of cadmium into the surfaces. Solutions and dilutions were prepared using ultrapure water (Merck Millipore Milli-Q™).

Breastmilk samples were allowed to melt at room temperature. To each 1 mL of breastmilk, 4 mL of 65% (v / v) HNO_3_ and 2 mL of 30% (v / v) hydrogen peroxide (H_2_O_2_) were added as previously described [16]. The mixture was allowed to digest in an environmental express hot-block digester (Environmental Express®­ HotBlock® 200, Cole-Parmer, Cambridgeshire, UK) at 120 º C for 40 min and then at 150 º C for 120 min [23]. The clear digestate was transferred to polypropylene tubes and adjusted to 50 mL using deionized water.

Breastmilk cadmium levels were determined against a calibration curve using inductively coupled plasma-mass spectrometry (ICP-MS, Perkin Elmer Elan 9000). The laboratories in which the analysis was conducted were ISO / IEC 17025:2017 certified.

Blank samples were prepared using HNO_3_ and H_2_O_2_ as well as a certified reference material that was prepared from skimmed milk powder. The limit of detection was calculated as threefold the standard deviation (SD) and the limit of quantification was calculated as tenfold the SD of 10 blank samples. The limit of detection was 0.0077 μg / L and the limit of quantification was 0.025 μg / L. There was no difference in the centration of cadmium detected and that in the certified value (0.0116 ± 0.0035 mg / kg vs. 0.0115 ± 0.0035 mg / kg). The recovery rate of cadmium from the reference material was 106%. The purity of the Aragon gas used in this study was 99.9990%. Thermo-Fisher Scientific's isotope ^111^Cd was used in this study.

### Theoretical daily exposure

Theoretical daily doses of cadmium via breastmilk were estimated using the quantified breastmilk cadmium concentrations and average daily consumption of breastmilk by the infants per 24 h [24]. Equation (2) was used to calculate the estimated daily intake of cadmium by the infants [25]:

2 $$EDI=\frac{\mathrm{C X }BMI per 24h}{\mathrm{Wt}}$$

where: C was the concentration of cadmium in breastmilk, BMI: breastmilk intake per 24 h, and Wt: weight of the infant.

### Data analysis

The data were entered into IBM SPSS for Windows, version 21.0. Differences in breastmilk cadmium levels were assessed using Student’s t-tests or analysis of variance (ANOVA). To control potentially confounding factors, the variables that were statistically significant in the Student’s t-tests or ANOVA were included in a multiple linear regression model. The goodness-of-fit was assessed using a significant R^2^. The absence of multicollinearity issues was determined using tolerance and variance inflation factor values. In this study, statistical significance was indicated by a *P*-value of < 0.05.

### Ethics

This study was conducted in adherence to the local and international guidelines regulating scientific research that involves human subjects and biological samples including those in the Declaration of Helsinki. Ethics approval was obtained from the Institutional Review Board of An-Najah National University (Protocol #: Oct. 2020 / 7). Approval was also obtained from the Office of Health Education. The lactating women provided written informed consent before any samples were collected.

## Results

### Characteristics of the lactating women

In this study, breastmilk samples were obtained from 256 lactating women. Demographic variables of the lactating women are provided in Table 1. The majority of the lactating women were less than 30 years old (64.1%), had 12 children (56.3%), age of child was six months and more (73.8%), were unemployed (93.0%), were non-smokers (78.9%), resided in rural areas (52.7%), lived within 500 m of an industrial area (57.4%), reported low monthly income (68.4%), did not attend a university (87.9%), lived far from disposals of wastes (92.2%), frequently used cosmetics (84.8%), used hair dyes less frequently (89.8%), lived far from paints shops (88.3%), did not live in a house with peeling / chipping paint (81.3%), and did not use vitamins frequently (61.7%). Of the lactating women, 17 (6.6%) lived in refugee camps.

**Table 1:** Demographic variables of the lactating women (*n* = *256*)

Variable	n	%
**Age (years)**		
< 30	164	64.1
≥ 30	92	35.9
**Number of children**		
≤ 2	144	56.3
> 2	112	43.8
**Age of child (months)**		
< 6	67	26.2
≥ 6	189	73.8
**Employment status**		
Unemployed	238	93.0
Employed	18	7.0
**Smoking status**		
Non-smoker	202	78.9
Smoker	54	21.1
**Place of residence**		
Rural	135	52.7
Urban	104	40.6
Camp of refugees	17	6.6
**Living in the vicinity of an industrial area**		
Far (more than 500 m)	109	42.6
Near (within 500 m)	147	57.4
**Household monthly income**		
Low	175	68.4
High	81	31.6
**Educational status**		
School	225	87.9
University	31	12.1
**Living in the vicinity of disposal of wastes**		
Far (more than 500 m)	236	92.2
Near (within 500 m)	20	7.8
**Use of cosmetics**		
Less frequent (occasionally / rarely)	39	15.2
More frequent (daily / almost daily)	217	84.8
**Use of hair dyes**		
Less frequent (never / once in a long while)	230	89.8
More frequent (usual)	26	10.2
**Distance to the closest paints shop**		
Far (more than 500 m)	226	88.3
Near (within 500 m)	30	11.7
**Peeling / chipping house paint**		
No	208	81.3
Yes	48	18.8
**Use of vitamins**		
Less frequent (never / once in a long while)	158	61.7
More frequent (usual)	98	38.3

### Breastmilk cadmium level

The mean breastmilk cadmium level in this study was 0.34 (SD: 0.33) μg / L and the mean EDI of cadmium via breastmilk was 0.059 (SD: 0.058) µg / kg per body weight / day. Breastmilk cadmium levels were not detected in 19 (7.4%) of the breastmilk samples. Of the breastmilk samples, 13 (5.1%) had cadmium levels above those reported as “normal” by the WHO. More samples contained cadmium levels above those reported as “normal levels” by the WHO in lactating women who were 30 years and older, were employed, were smokers, resided in urban areas or camps of refugees, had high household income, lived close to disposals of wastes, frequently used hair dyes, lived close to paints shops, and did not use vitamins frequently. Details of these associations are shown in Supplementary Table S2.

In this study, breastmilk cadmium levels and EDI were significantly higher among lactating women who were 30 years and older, had more than two children, were employed, were smokers, resided in urban areas or camps of refugees, lived close to an industrial area, lived close to disposals of wastes, frequently used cosmetics, frequently used hair dyes, lived close to paints shops, lived in a house with peeling / chipping paint, and did not use vitamins frequently. On the other hand, variables like age of child, monthly income, and educational status were not significantly associated with breastmilk cadmium levels. Details of these associations are shown in Table 2.

**Table 2:** Breastmilk cadmium levels and estimated daily intake of cadmium according to participant characteristics

			**Breastmilk cadmium level (μg / L)**				**Estimated daily intake (µg / kg per body weight / day)**			
**Variable**	**n**	**%**	**Mean**	**SD**	**DF**	***p*-value**	**Mean**	**SD**	**DF**	***p*-value**
**Age (years)**										
< 30	164	64.1	0.30	0.02	254	0.02	0.05	0.05	254	0.03
≥ 30	92	35.9	0.40	0.04			0.07	0.07		
**Number of children**										
≤ 2	144	56.3	0.29	0.02	254	0.04	0.05	0.05	254	0.04
> 2	112	43.8	0.40	0.04			0.07	0.07		
**Age of child (months)**										
< 6	67	26.2	0.31	0.04	254	0.41	0.05	0.05	254	0.35
≥ 6	189	73.8	0.35	0.02			0.06	0.06		
**Employment status**										
Unemployed	238	93.0	0.31	0.02	254	0.00	0.05	0.05	254	0.00
Employed	18	7.0	0.74	0.15			0.13	0.11		
**Smoking status**										
Non-smoker	202	78.9	0.29	0.02	254	0.00	0.05	0.05	254	0.00
Smoker	54	21.1	0.51	0.07			0.09	0.08		
**Place of residence**										
Rural	135	52.7	0.22	0.02	253^a^	0.00	0.04	0.00	253^a^	0.00
Urban	104	40.6	0.43	0.02			0.07	0.00		
Refugee camp	17	6.6	0.77	0.16			0.13	0.03		
**Living in the vicinity of an industrial area**										
Far (more than 500 m)	109	42.6	0.28	0.04	254	0.01	0.05	0.07	254	0.01
Near (within 500 m)	147	57.4	0.38	0.02			0.07	0.05		
**Household monthly income**										
Low	175	68.4	0.32	0.21	254	0.09	0.05	0.04	254	0.08
High	81	31.6	0.39	0.50			0.07	0.09		
**Educational status**										
School	225	87.9	0.33	0.02	254	0.23	0.06	0.05	254	0.23
University	31	12.1	0.41	0.08			0.07	0.08		
**Living in the vicinity of a main disposal of wastes**										
Far (more than 500 m)	236	92.2	0.29	0.01	254	0.00	0.05	0.03	254	0.00
Near (within 500 m)	20	7.8	0.97	0.16			0.17	0.13		
**Use of cosmetics**										
Less frequent (occasionally / rarely)	39	15.2	0.24	0.33	254	0.04	0.04	0.06	254	0.04
More frequent (daily / almost daily)	217	84.8	0.36	0.33			0.06	0.06		
**Use of hair dyes**										
Less frequent (never / once in a long while)	230	89.8	0.31	0.27	254	0.00	0.05	0.05	254	0.00
More frequent (usual)	26	10.2	0.58	0.61			0.10	0.11		
**Distance to the closest paint shop**										
Far (more than 500 m)	226	88.3	0.32	0.02	254	0.00	0.06	0.05	254	0.00
Near (within 500 m)	30	11.7	0.50	0.11			0.09	0.11		
**Peeling / chipping house paint**										
No	208	81.3	0.32	0.02	254	0.04	0.06	0.05	254	0.03
Yes	48	18.8	0.43	0.06			0.07	0.08		
**Use of vitamins**										
Less frequent (never / once in a long while)	158	61.7	0.41	0.39	254	0.00	0.07	0.07	254	0.00
More frequent (usual)	98	38.3	0.23	0.12			0.04	0.02		

^a^Within groups

*DF* Degree of freedom

When these variables were included in a multiple linear regression model, being a smoker, living in camps of refugees, living close to an industrial area, living close to disposals of wastes, frequent use of cosmetics, frequent use of hair dyes, living close to paints shops, living in a house with peeling / chipping paint, and not using vitamins predicted higher levels of breastmilk cadmium levels and higher EDI. Details of the multiple linear regression model are shown in Table 3.

**Table 3:** Predictors of higher breastmilk cadmium levels

	**Variable**	**Unstandardized coefficients**	**SE**	**Standardized coefficients**	**t**	***p*-value**
**Breastmilk cadmium level**	Age	0.01	0.03	0.01	0.27	0.79
	Number of children	0.03	0.03	0.04	0.94	0.35
	Employment status	0.09	0.06	0.07	1.52	0.13
	Smoking status	0.09	0.04	0.11	2.35	0.02
	Place of residence	0.18	0.03	0.34	7.15	0.00
	Living in the vicinity of an industrial area	0.08	0.03	0.12	2.56	0.01
	Living in the vicinity of a main disposal of wastes	0.55	0.06	0.45	9.26	0.00
	Use of cosmetics	0.11	0.04	0.12	2.86	0.00
	Use of hair dyes	0.07	0.05	0.06	1.34	0.18
	Distance to the closest paints shop	0.22	0.06	0.22	3.57	0.00
	Peeling / chipping house paint	0.16	0.05	0.19	3.17	0.00
	Use of vitamins	-0.10	0.03	-0.14	-3.17	0.00
**Estimated daily intake**	Age	0.00	0.01	0.01	0.15	0.88
	Number of children	0.00	0.01	0.04	0.91	0.37
	Employment status	0.02	0.01	0.07	1.54	0.12
	Smoking status	0.02	0.01	0.11	2.39	0.02
	Place of residence	0.03	0.00	0.34	7.07	0.00
	Living in the vicinity of an industrial area	0.01	0.01	0.12	2.52	0.01
	Living in the vicinity of a main disposal of wastes	0.10	0.01	0.45	9.33	0.00
	Use of cosmetics	0.02	0.01	0.12	2.79	0.01
	Use of hair dyes	0.01	0.01	0.06	1.37	0.17
	Distance to the closest paints shop	0.04	0.01	0.22	3.61	0.00
	Peeling / chipping house paint	-0.03	0.01	-0.19	-3.15	0.00
	Use of vitamins	-0.02	0.01	-0.14	-3.13	0.00

*SE* Standard error, *t* t-statistics

## Discussion

Chronic exposure to cadmium-contaminated breast milk can lead to buildup that would be excreted from the infant’s body at a slow rate [26]. Infants are vulnerable to cadmium due to insufficient antioxidant systems such as glutathione to reduce cadmium toxicity. Therefore, assessing breastmilk cadmium levels and identifying predictors of high breastmilk cadmium levels and high infant exposure would help inform biofluid monitoring decisions, safe breastfeeding, and counseling. For the first time, breastmilk samples were assessed for cadmium levels. Additionally, predictors of high breastmilk cadmium levels and high exposure were identified in Palestine as a resource-limited country.

Previous studies have reported variable breastmilk cadmium levels among breastfeeding women from Iran, Morocco, Spain, and Norway [4, 5, 15–17]. It is noteworthy that quantification of cadmium levels could be highly dependent on the sensitivity of the methods used. Additionally, the demographic and exposure characteristics of the women who were included in this study could be different from those who were included in the previous studies that were conducted elsewhere. In this study, cadmium levels could be quantified in 92.6% of the breastmilk samples. Comparatively, cadmium was detected in 99% of the breastmilk samples collected from the region of Ankara [27]. However, cadmium was detected in 38% of the breastmilk samples collected from Spain [5]. Moreover, only 5.1% of the breastmilk samples in this study had cadmium levels above the cutoff used by the WHO compared to 30% of the breastmilk samples in Morocco [16]. Taken together, these findings might indicate differential dietary and environmental maternal exposure to cadmium sources. Probably, the low breastmilk cadmium levels reported in this study could be explained by the relatively recent urbanization and low industrialization of the different regions of Palestine.

In this study, breastmilk cadmium levels were higher among lactating women who were older than 30 years. Cadmium levels accumulate over time, notably, when excretion rates are slow [26]. Probably, lactating women who were older than 30 years have a buildup of cadmium stores. It is well-established that cadmium can be transferred into breast milk [28, 29]. Eventually, breastmilk obtained from older lactating women contained higher cadmium levels that could be transferred to nourishing infants. Similarly, breastmilk cadmium levels were higher in lactating women who were exposed to sources of cadmium including: living in a refugee camp, close to industrial areas disposal of wastes, paints shops, or living in a house with chipping or peeling paint. These findings were not surprising as disposal of wastes, fossil fuel burning, and incinerators were important sources of cadmium [7]. Therefore, the breastmilk samples obtained from lactating women who lived in less hygienic conditions and closer to sources of contamination had higher cadmium levels.

The findings of this study showed that breastmilk cadmium levels were higher among lactating women who smoked tobacco. The findings reported in this study were consistent with those reported in previous studies in which tobacco smoking was a source of exposure to cadmium [9, 30]. Taken together, these findings indicate that gynecologists, pediatricians, midwives, nurses, breastfeeding counselors, and other providers of antenatal and postnatal healthcare services should consider counseling current and future breastfeeding women about the deleterious effects of tobacco smoking.

In this study, frequent use of cosmetics and hair dyes was associated with higher breastmilk cadmium levels. Studies conducted in different countries have shown that some cosmetic products contained heavy metals including cadmium in concentrations above those determined to be safe by the health regulatory authorities [31–33]. A study in Iran showed that serum cadmium levels were higher among women who reported frequent facial cosmetics use [32]. Cadmium levels were significantly and positively correlated with the duration of use of cosmetics. Similarly, cadmium was detected in hair dyes and other hair coloring cosmetics [34, 35]. A previous study quantified cadmium in canned food products in Palestine [8]. Taken together, these findings indicate the regulatory authorities should adopt a stricter control strategy to reduce / prevent the availability of these products to consumers. Additionally, authorities should plan and implement surveillance programs to determine cadmium and other harmful heavy metals in cosmetics and beauty products. Implementing these measures and prohibiting the marketing of these harmful products might reduce cadmium levels in breast milk of lactating women.

In this study, breastmilk cadmium levels were significantly higher among women who were employed. Probably, employed women were more likely to be older than 30 years, to smoke tobacco, use cosmetic products, and be occupationally exposed to sources of cadmium.

The findings of this study showed that breastmilk cadmium levels were significantly lower among the lactating women who reported frequent use of vitamins. Previous studies showed that some vitamins were negatively correlated with cadmium levels. A recent study showed that blood cadmium levels were inversely associated with vitamin D levels in Chinese women [36]. Other studies in animals showed that supplementation of vitamin C reduced cadmium levels in different tissues including kidneys, liver, testicles, and muscles [37]. Additionally, vitamins C and E were shown to reduce reactive oxygen species and have protective effects against cadmium exposure [38–40]. Taken together, these findings may indicate the need to encourage women of childbearing age to consume the recommended amounts of vitamins on regular basis.

### Strengths and limitations

The findings of this study might be interpreted after considering the following strengths and limitations. This is the first study to determine breastmilk cadmium levels in lactating women in Palestine. Monitoring levels of harmful heavy metals in breastmilk and other biological fluids could be informative to decision and policymakers in resource-limited countries. Second, a large sample size was used in this study. Additionally, the lactating women from whom breastmilk samples were obtained were recruited from different primary and maternity healthcare clinics in Palestine. The sample included primiparas and multiparas. Moreover, the lactating women were diverse in terms of demographic variables and exposure to sources of cadmium. The large sample size and diversity of the women should have improved the representativeness of the entire population of lactating women and the external validity of the findings reported in this study. Third, a validated and robust ICP-MS method was used to assess breastmilk cadmium levels in this study. Compared to other methods like atomic absorption spectroscopy, ICP-MS is more sensitive in quantifying elements in biological fluids including breastmilk. Fourth, theoretical infant exposure to cadmium via breastmilk was estimated using an acceptable approach. Fifth, this study was conducted and reported in adherence to the STROBE statement. Adherence to such consensus-based guidelines should have improved congruence and transparency in reporting the findings. This might also promote comparability of the findings reported elsewhere.

However, the following limitations were associated with this study. First, the breastmilk samples that were analyzed in this study were foremilk only. It would have been interesting to analyze and compare foremilk, hindmilk, and whole milk samples. Second, a cross-sectional design was used in this study. Compared to interventional studies, observational studies are limited by approach. It would have been more interesting if the women with high breastmilk cadmium levels were given treatment over an extended period or in a time series. Third, blood or plasma samples from infants were not obtained and assessed for cadmium levels. It would have been interesting to quantify and correlate cadmium levels in breast milk and blood or plasma of infants. Fourth, the EDI would only apply when infants are exclusively breastfed. Assessing cadmium levels in the different forms of infant formulas available in the local markets could have provided valuable data. Finally, we did not quantify cadmium levels in drinking water and foods consumed by the women who provided breastmilk samples in this study. Identifying sources of exposure to cadmium could inform measures to reduce such exposure.

## Conclusion

Breastmilk cadmium levels were quantified among lactating women in a resource-limited country. Additionally, theoretical infant exposure to cadmium via breastmilk was also estimated. The breastmilk cadmium levels and infant exposure were predicted by maternal exposure to sources of cadmium like tobacco smoking, living in camps of refugees, living close to an industrial area, living close to disposal of wastes, frequent use of cosmetics, frequent use of hair dyes, living close to paints shops, living in a house with peeling / chipping paint, and not consuming vitamins. The findings reported in this study are valuable to gynecologists, pediatricians, breastfeeding advisors, and other providers of antenatal and postnatal healthcare services. More studies are still needed to plan and implement measures to reduce breastmilk cadmium levels and exposure of infants to cadmium via breastmilk.

## Supplementary Information

**Additional file 1.**

## Data Availability

All data relevant to this study were included in the results section of this manuscript.

## Abbreviations

BMI: Body mass index
CI: Confidence interval
DF: Degrees of freedom
EDI: Estimated daily intake
ICP-MS: Inductively coupled plasma mass spectrometry
SD: Standard deviation
STROBE: Strengthening the reporting of observational studies in epidemiology
WHO: World Health Organization
Wt: Weight
