# Supplementary material for: Breastmilk cadmium levels and estimated infant exposure: a multicenter study of associated factors in a resource-limited country
Source: Int Breastfeed J. 2023 Jul 28;18:36. doi: 10.1186/s13006-023-00574-0 (PMC10375743; doi:10.1186/s13006-023-00574-0)
Supplement: Supplementary file 1 — Additional file 1. [file 13006_2023_574_MOESM1_ESM.docx]

**Supplementary materials for**

**Breastmilk cadmium levels and estimated infant exposure: A multicenter study of associated factors in a resource-limited country**

Ramzi Shawahna^1,2*^, Rana Saleh^3^, Lina Owiwi^3^, Iyad Maqboul^3,4^, Hatim Hijaz^3,4^, Mohammad Jaber^3,4^

^1^Department of Physiology, Pharmacology and Toxicology, Faculty of Medicine and Health Sciences, An-Najah National University, Nablus, Palestine

^2^Clinical Research Center, An-Najah National University Hospital, Nablus 44839, Palestine

^3^Department of Medicine, Faculty of Medicine and Health Sciences, An-Najah National University, Nablus, Palestine

^4^An-Najah National University Hospital, Nablus 44839, Palestine

**^*^Correspondence:**

Ramzi Shawahna, PhD, Department of Physiology, Pharmacology and Toxicology, Faculty of Medicine & Health Sciences, New Campus, Building: 19, Office: 1340, An-Najah National University, P.O. Box 7, Nablus, Palestine

Phone: + (970) 923 45113 ext 2772

Phone: + (970) 92349739

Email: [ramzi_shawahna@hotmail.com](mailto:ramzi_shawahna@hotmail.com)

**Supplementary Table S1:** The questionnaire

| Please indicate your age in years |  |  |  |
| --- | --- | --- | --- |
| How many children do you have? |  |  |  |
| What is the age of the child you are currently breastfeeding in months? |  |  |  |
| Are you employed? | □ Unemployed | □ Employed |  |
| Do you smoke tobacco? | □ No | □ Yes |  |
| Where do you live? | □ Rural area | □ Urban area | □ Camp of refugees |
| How would you rate your household income? | □ Low | □ High |  |
| What is your educational level? | □ School | □ University |  |
| Please indicate the distance from your house to the closest industrial area in meters |  |  |  |
| Please indicate the distance from your house to the closest disposal of wastes |  |  |  |
| Please indicate the distance from your house to the closest paints shop |  |  |  |
| How often do you use cosmetics? | □ Occasionally/rarely | □ Daily/almost daily |  |
| How often do you use hair dyes? | □ Never/once in a long while | □ Usual use |  |
| Do you live in a house whose paint is peeling/chipping? | □ No | □ Yes |  |
| How often do you use vitamins? | □ Never/once in a long while | □ Usual use |  |

**Supplementary Table S2:** Associations between demographic variables of the lactating women and breastmilk cadmium levels

|  | **Breastmilk cadmium level** | | | |  |  |
| --- | --- | --- | --- | --- | --- | --- |
|  | **< 1 μg/L** | | **≥ 1 μg/L** | |  |  |
| **Variable** | **n** | **%** | **n** | **%** | **Chi-square** | **p-value** |
| **Age (years)** |  |  |  |  |  |  |
| < 30 | 160 | 62.5 | 4 | 1.6 | 6.27 | 0.016 |
| ≥ 30 | 83 | 32.4 | 9 | 3.5 |  |  |
| **Number of children** |  |  |  |  |  |  |
| ≤ 2 | 140 | 54.7 | 4 | 1.6 | 3.61 | 0.083 |
| > 2 | 103 | 40.2 | 9 | 3.5 |  |  |
| **Lactating since (months)** |  |  |  |  |  |  |
| < 6 | 64 | 25.0 | 3 | 1.2 | 0.07 | 1.000 |
| ≥ 6 | 179 | 69.9 | 10 | 3.9 |  |  |
| **Employment status** |  |  |  |  |  |  |
| Unemployed | 232 | 90.6 | 6 | 2.3 | 45.74 | < 0.001 |
| Employed | 11 | 4.3 | 7 | 2.7 |  |  |
| **Smoking status** |  |  |  |  |  |  |
| Non-smoker | 196 | 76.6 | 6 | 2.3 | 7.14 | 0.008 |
| Smoker | 47 | 18.4 | 7 | 2.7 |  |  |
| **Place of residence** |  |  |  |  |  |  |
| Rural | 133 | 52.0 | 2 | 0.8 | 30.45 | < 0.001 |
| Urban | 101 | 39.5 | 3 | 1.2 |  |  |
| Camp of refugees | 9 | 3.5 | 8 | 3.1 |  |  |
| **Living in the vicinity of an industrial area** |  |  |  |  |  |  |
| Far (more than 500 m) | 103 | 40.2 | 6 | 2.3 | 0.07 | 1.000 |
| Near (within 500 m) | 140 | 54.7 | 7 | 2.7 |  |  |
| **Household monthly income** |  |  |  |  |  |  |
| Low | 172 | 67.2 | 3 | 1.2 | 12.93 | 0.001 |
| High | 71 | 27.7 | 10 | 3.9 |  |  |
| **Educational status** |  |  |  |  |  |  |
| School | 215 | 84.0 | 10 | 3.9 | 1.54 | 0.377 |
| University | 28 | 10.9 | 3 | 1.2 |  |  |
| **Living in the vicinity of a main disposal of wastes** |  |  |  |  |  |  |
| Far (more than 500 m) | 232 | 90.6 | 4 | 1.6 | 71.45 | < 0.001 |
| Near (within 500 m) | 11 | 4.3 | 9 | 3.5 |  |  |
| **Use of cosmetics** |  |  |  |  |  |  |
| Less frequent (occasionally/rarely) | 38 | 14.8 | 1 | 0.4 | 0.60 | 0.698 |
| More frequent (daily/almost daily) | 205 | 80.1 | 12 | 4.7 |  |  |
| **Use of hair dyes** |  |  |  |  |  |  |
| Less frequent (never/once in a long while) | 222 | 86.7 | 8 | 3.1 | 11.98 | 0.005 |
| More frequent (usual) | 21 | 8.2 | 5 | 2.0 |  |  |
| **Distance to the closest paints shop** |  |  |  |  |  |  |
| Far (more than 500 m) | 218 | 85.2 | 8 | 3.1 | 9.43 | 0.010 |
| Near (within 500 m) | 25 | 9.8 | 5 | 2.0 |  |  |
| **Peeling/chipping house paint** |  |  |  |  |  |  |
| No | 198 | 77.3 | 10 | 3.9 | 0.17 | 0.715 |
| Yes | 45 | 17.6 | 3 | 1.2 |  |  |
| **Use of vitamins** |  |  |  |  |  |  |
| Less frequent (never/once in a long while) | 145 | 56.6 | 13 | 5.1 | 8.46 | 0.006 |
| More frequent (usual) | 98 | 38.3 | 0 | 0.0 |  |  |
